# Damage Identification in Cement-Based Structures: A Method Based on Modal Curvatures and Continuous Wavelet Transform

**DOI:** 10.3390/s23229292

**Published:** 2023-11-20

**Authors:** Gloria Cosoli, Milena Martarelli, Alessandra Mobili, Francesca Tittarelli, Gian Marco Revel

**Affiliations:** 1 Department of Industrial Engineering and Mathematical Sciences, Marche Polytechnic University, 60131 Ancona, Italy; m.martarelli@staff.univpm.it (M.M.); gm.revel@staff.univpm.it (G.M.R.); 2 Department of Materials, Environmental Sciences and Urban Planning, Marche Polytechnic University, 60131 Ancona, Italy; a.mobili@staff.univpm.it (A.M.); f.tittarelli@staff.univpm.it (F.T.); 3 Institute of Atmospheric Sciences and Climate, National Research Council (ISAC-CNR), 40129 Bologna, Italy

**Keywords:** cement-based structure, modal analysis, modal curvature, damage identification, continuous wavelet transform, Structural Health Monitoring

## Abstract

Modal analysis is an effective tool in the context of Structural Health Monitoring (SHM) since the dynamic characteristics of cement-based structures reflect the structural health status of the material itself. The authors consider increasing level load tests on concrete beams and propose a methodology for damage identification relying on the computation of modal curvatures combined with continuous wavelet transform (CWT) to highlight damage-related changes. Unlike most literature studies, in the present work, no numerical models of the undamaged structure were exploited. Moreover, the authors defined synthetic damage indices depicting the status of a structure. The results show that the I mode shape is the most sensitive to damages; indeed, considering this mode, damages cause a decrease of natural vibration frequency (up to approximately −67%), an increase of loss factor (up to approximately fivefold), and changes in the mode shapes morphology (a cuspid appears). The proposed damage indices are promising, even if the level of damage is not clearly distinguishable, probably because tests were performed after the load removal. Further investigations are needed to scale the methodology to in-field applications.

## 1. Introduction

Structural Health Monitoring (SHM) technologies are pivotal for the management of cement-based structures and infrastructures, which can be frequently subjected to destructive factors causing damage to the structure itself. Indeed, both the costs and life cycle of structures and infrastructures can be optimized through the regular monitoring of relevant parameters, providing a broad picture of the health status of the supervised structure, and avoiding rapid damage propagation. Given the relevant costs of inspection, maintenance, and intervention operations, the SHM techniques can play an essential role in assessing damages or even predicting their occurrence. The advantages concerning inspections are unquestionable, given the prompt identification of structural issues and the consequent planning of deeper inspection operations, providing an invaluable contribution, especially for critical structures [1]. Moreover, monitoring tools also allow for the inclusion of environmental parameters in the evaluation, properly considering their effect on the measured signals, varying with these factors [2,3]. In this regard, Ietka et al. [4] highlighted the correlation between temperature and displacement in a bridge case study. However, even with all the potentialities of SHM strategies, at present, it is not easy to widely diffuse the installation of a SHM system on buildings or infrastructures, since the potential economic benefit is not so straightforward to final users [5,6], despite the possibility of achieving completely automated monitoring systems through the exploitation of sophisticated smart sensors and also Artificial Intelligence (AI) tools [7,8].

In the last decades, several different technologies have been exploited to this aim, each showing pros and cons; among them, it is worthy to cite self-sensing/monitoring techniques (where electrical impedance sensors are strictly related to materials with conductive additions enhancing their self-sensing/monitoring capability [9]) and smart sensor networks exploiting deep learning computing tools [1], acoustic sensors [10], piezoelectric transducers [11], non-contact vibration sensors [12], accelerometers [13], etc. Different time scales can be considered for diagnosis, also in (near) real-time, hence useful for emergency operations [14] and also in combination with early warning systems [15,16]. Sometimes static analyses have been exploited, for example with parameter identification to identify damages in frame structures [17] or stiffness separation method on truss structures [18]. Vibrational analysis (in particular, operational modal analysis coupled with proper signal processing techniques [19]) can depict the dynamical performance of a structure and reflects eventual changes due to disrupting events [20] (e.g., earthquakes [21]) or deterioration processes (e.g., contaminants penetration). In fact, in case of damage, the dynamic response of the structure is different with respect to the undamaged status [22]. Mass, stiffness, and damping values are altered, reflecting in changes in terms of modal parameters (i.e., natural frequency and loss factor) as well as of mode shapes and related modal curvatures [23,24,25] (also known as rotational modes [26]), which are the second derivative of mode shapes, usually computed according to the central difference approximation method [22]. Sometimes, the flexibility matrix has also been considered for damage identification purposes, being more effective at low frequency with respect to the stiffness matrix [25]. Modal curvatures are generally more affected by singularities concerning the corresponding mode shapes; in fact, the curvature is related to the structure stiffness, which in turn varies with the presence of cracks (i.e., local damages [27]). Therefore, the local stiffness reduction due to the formation of a crack makes the curvature lose continuity [28]. Impact tests have been extensively exploited in the literature for damage identification in concrete structures [29]; however, a significant number of issues can occur in experimental conditions, making it difficult to estimate the second derivative of mode shapes with high accuracy [30], especially in noisy environments, where dedicated processing techniques are needed [31]. Moreover, while on concrete plain structures, the damage can be considered as a concentrated defect, in reinforced concrete elements the intrinsic heterogeneity leads to non-linear behaviours, which cause diffuse cracking phenomena, possibly masking the effect of singularities. This behaviour is also enhanced by the quasi-static nature of the applied loads (e.g., monotonic or cycling loading). A common approach to analyze this type of damage is the evaluation of the natural frequency reduction, which appears very evident in the initial cracking phase (up to 25–55% in the yield state [32,33]). Its measurement appears quite straightforward, hence this method can be widely exploited for damage localization [34]. Moreover, adding virtual mass to a structure can be effective in the enhancement of sensitivity toward damages [35]. On the other hand, damping ratios are challenging to measure, being not so regular [36] and consequently not commonly exploited for damage identification.

Several studies consider numerical models to discuss the relationship between the presence of damages and the related changes in modal parameters. In fact, since the information related to the intact structure is frequently unavailable and many approaches are based on a comparison with this time, numerical models can fill this gap. However, there are inevitable simplifications in the modelling procedures, not suffering from noise and disturbing sources, differently from experimental cases. Indeed, to effectively deal with noisy data typical of experimental campaigns, different routes can be pursued [28]: (i) design an optimal sampling in order to reduce errors when computing modal curvatures [37]; (ii) exploit suitable filtering techniques (e.g., Laplacian scheme [38] or Teager energy operator and wavelets [39]); (iii) use effective post processing techniques (e.g., fuzzy logic [40,41]).

Moreover, many studies in the literature define damage-related indices to investigate the structural health status of a structure concisely. Meruane et al. [42] developed a mathematical framework for damage detection in steel-concrete composites subjected to impact tests: they studied the changes in stiffness due to damage occurrence and evaluated the correlation with the initial condition through the modal assurance criterion (MAC). A damage index based on curvatures (i.e., the absolute difference in curvature between damaged and intact structures) was synthesized to depict the distribution of damages. In fact, sharp changes in modal curvatures are caused by structural damage. Indeed, the absolute difference in terms of modal curvatures between damaged and undamaged states is quite widely exploited in the literature [43]; Pranno et al. [22] defined a Curvature Damage Factor (CDF), averaging the differences in modal curvatures considering a certain number of mode shapes in damaged/undamaged configurations; also in this case, a simulation-based damage detection approach was proposed to evaluate the stiffness degradation with progressive damage occurrence. They also underlined the relevance of the inverse eigenvalue sensitivity method [44] for identifying damages through variations in terms of natural vibration frequencies. Jahangir et al. [45] proposed different damage indices based on single, double, and triple damage scenarios represented in a numerical model; they calculated the energy associated with the wavelet coefficients through the Shannon entropy and defined two indices able to localize the damage and assess its severity. Indeed, wavelets have been demonstrated to be sensitive to the presence of damages (e.g., Haar and Gabor wavelets for crack detection [46]) and represent a powerful tool to be exploited in this context, along with numerical methods. Masciotta and Pellegrini [47] proposed an index based on the imaginary component of mode shapes in different damage conditions; in particular, they considered the difference in the imaginary part (normalized with respect to the initial average imaginary content) and multiplied it by the ratio between natural frequency in damaged and undamaged conditions, evidencing the higher sensitivity to damage occurrence with respect to the real component. Bayissa et al. [48] exploited continuous wavelet transform (CWT) to derive the energy distribution in the time-frequency domain and transform the wavelet coefficients into damage identification parameters.

Some studies investigated also the damage severity, which can be related to the energy of wavelet coefficients computed in correspondence with the diverse degrees of freedom (DOFs) [45]. Moreover, Jahangir et al. [49] evidenced that wavelets are efficient in eliminating noise that could contaminate curvatures and prevent correct damage detection through singularity identification. Starting from the details signal provided by the wavelet analysis, they also proposed a normalized damage index to be computed for each DOF, to localize the damage according to a specific threshold. The use of wavelets can be necessary to highlight discontinuities also in the presence of relatively small damages, which could be easily masked by noise; however, wavelets also inherit global fluctuations, therefore sometimes they are not sufficient. In those cases, the Teager energy operator can be exploited to intensify local singularities while removing global trend fluctuations; Sha et al. [50] exploited this approach and proposed a damage index based on data fusion of multiple mode shapes analysed through wavelets and Teager energy operator techniques.

Also, the variability of modal parameters must be considered; indeed, both environmental and material-intrinsic factors can make natural vibration frequency change and this needs to be distinguished from variations caused by a damage occurrence. Anastasopoulos et al. [51] found the same sensitivity of natural frequency towards damage and temperature; Maes et al. [52] proposed to use of the Principal Component Analysis (PCA) to remove the variations due to natural factors when validating vibration-based SHM on a retrofitted railway bridge. In general, repeatability tests should be performed to evaluate the robustness of a specific method.

The information deriving from a modal analysis could also represent an added value to be exploited in AI-based techniques for SHM [53,54]. Indeed, SHM-related data processing can provide multi-domain information supporting decision-making strategies [55] and really making a difference, even more through the exploitation of machine learning (ML) algorithms, especially the unsupervised ones, not requiring fully labelled data [55], or deep learning approaches [56]. However, for this aim great amounts of data are needed for proper model training and this goes beyond the scope of this work.

This manuscript aims at:

- Evaluating the changes in terms of modal parameters of scaled concrete beams subjected to loading tests leading to cracking phenomena.
- Analyzing the modal curvatures also through continuous wavelet transform.
- Proposing damage indices considering both the curvature change and the CWT-based analysis.
- Evaluating the sensitivity of the results with respect to data processing parameters.

Scaled concrete beams were manufactured with a self-sensing mix design; loading tests were performed at different levels by using a mechanical press. After each loading phase, an impact test was performed to assess the dynamic behaviour of the concrete elements. Modal analysis was conducted, and modal curvatures were analyzed, also exploiting CWT. Finally, different damage indices were proposed.

The paper is organised as follows: Section 2 describes the materials and methods employed in the study, starting with the description of the concrete specimens and their mix design and the load tests performed. Then, the techniques adopted for modal analysis and modal curvature computation are reported, together with the definition of damage-related indices. Hence, the methods for sensitivity analysis towards processing parameters are detailed. The results are reported and discussed in Section 3. Finally, the authors draw the conclusions in Section 4, where also possible future developments are depicted.

## 2. Materials and Methods

Concrete specimens were manufactured according to the mix design reported in Table 1; the water/cement (w/c) ratio was equal to 0.50 by mass and the workability class was S5 assessed through the slump Abrams cone. It is worth noting that the selection (and dosages) of conductive carbon-based additions, namely recycled carbon fibres (RCF) and biochar (BCH), was made according to the findings of a previous study [9] (furthermore, the developed mix-design and the related measurement system have already been patented—“Eco-compatible and self-sensing mortar and concrete compositions for manufacturing reinforced and non-reinforced constructive elements, related construction element and methods for the realization of self-monitorable building structures”, patent n. 102020000022024); these materials enhance the self-sensing capabilities of cement-based elements, but the related analysis goes beyond the scope of this paper.

A concrete mixer was used for casting and the following steps were accomplished:

- Mixing of sand and intermediate/coarse gravels (2 min).
- Addition of cement and further mixing (2 min).
- Addition of BCH and further mixing (7 min).
- Addition of RCF and further mixing (2 min).
- Water addition and further mixing (10 min).
- Pouring of fresh mix in moulds.

**Figure 1:**
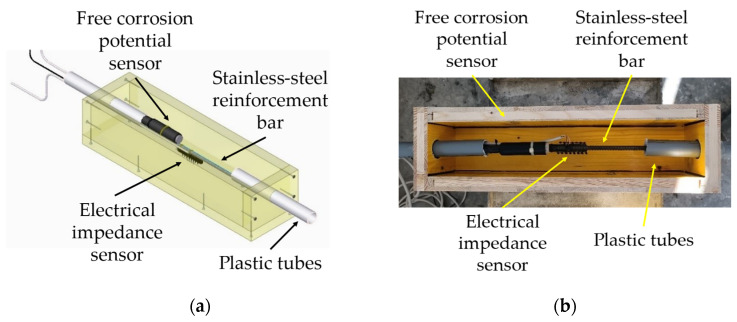
Layout of the sensorized concrete specimens: (**a**) Design; (**b**) Mold for casting phase.

A total of 12 prismatic (10 cm × 10 cm × 50 cm) specimens were cast; they were reinforced with a corrugated stainless-steel rebar placed in the middle. Prismatic specimens were divided as follows:

- n. 6 sensorized specimens: sensors for the measurement of electrical impedance and free corrosion potential were embedded in the specimens for SHM purposes (both beyond the scope of this article, but very important to continuously monitor the health status of the material). Plastic tubes were employed for easing the cable routing; they require particular attention since they inevitably contribute to the determination of the element dynamic behaviour. The layout of the specimens is reported in Figure 1.
- n. 6 non-sensorized specimens: these were manufactured to evaluate the effect of the embedded sensors on the dynamic behaviour of the elements (rigidity should be affected by sensors, representing discontinuities in the material) and the consequent modal parameters. Half of them were dedicated to the assessment of flexural strength according to the EN 12390-5 standard [57]; the obtained value was relevant for the design of the loading tests to be performed on the concrete beams.

After the standard curing period (i.e., 28 days) in environmental conditions, stainless-steel washers were installed with a bicomponent resin to serve as bases for the positioning of accelerometers for the vibrational analysis. Hence, the specimens were subjected to loading tests. Increasing load levels were determined depending on the mechanical resistance preliminarily measured on dedicated specimens.

**Figure 2:**
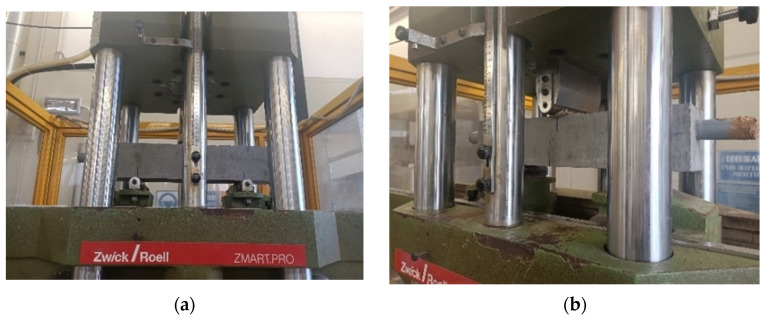
Mechanical press: (**a**) Frontal view; (**b**) Angular view.

The loading tests were executed through a mechanical press (Zwick Roell, maximum load: 600 kN, Figure 2). The concrete specimen was positioned on two pins (i.e., pinned conditions) at 30 cm from each other (i.e., 10 cm from the beam ends); the load was applied on the specimen centre, with a velocity of 0.1 mm/min. Three different loads were applied (and the corresponding test times were named as reported hereafter), namely:

- 90% of the fracture load assessed on dedicated specimens (*t*1).
- Fracture load (i.e., the load at which the first crack forms), specific for the specimen under test (*t*2).
- The load at which the crack aperture is approximately 1 mm (*t*3).

The initial time, when the specimen was intact, was called *t*0.

**Figure 3:**
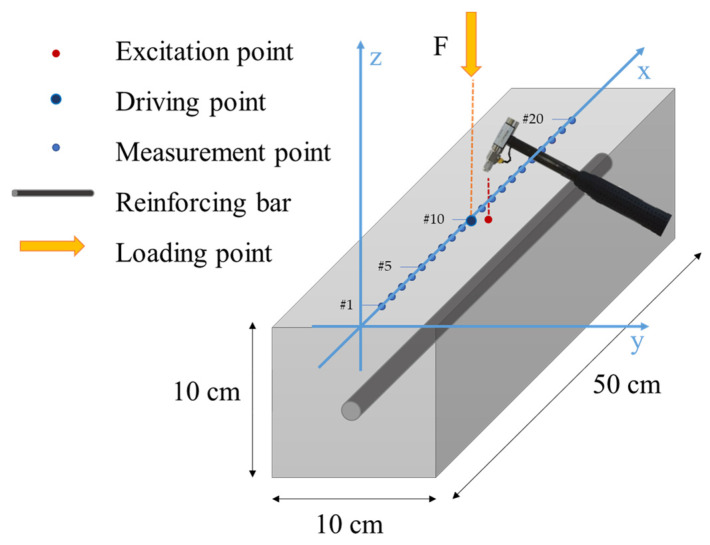
Specimen configuration for the impact test.

At *t*0 and after each load trial, a vibrational analysis was performed; an impact hammer (PCB 086 B04, PCB Piezotronics, Depew, NY, USA) was employed to excite the structure (force given in −z direction, see Figure 3), hence the structural response was measured through accelerometers (measuring on z direction) positioned along the specimen mid-line; 20 DOFs were considered, with an interaxle spacing of 2.5 cm from each other (Figure 3). The DOF #10 can be considered the driving point, given that the energy path from the excitation point is short enough (<5 mm, according to the sensor dimensions).

**Figure 4:**
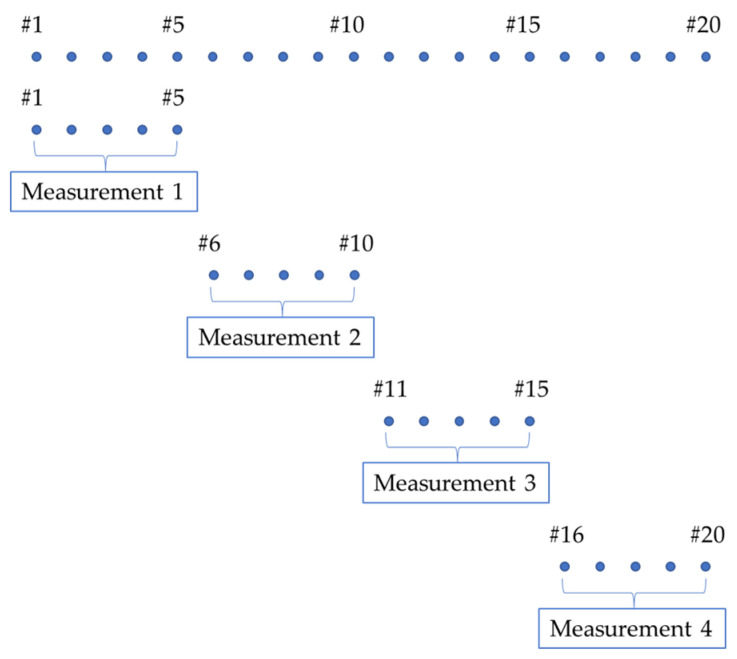
Sequence of measurements with 5 accelerometers; 4 measurements are necessary to cover the whole specimen.

**Figure 5:**
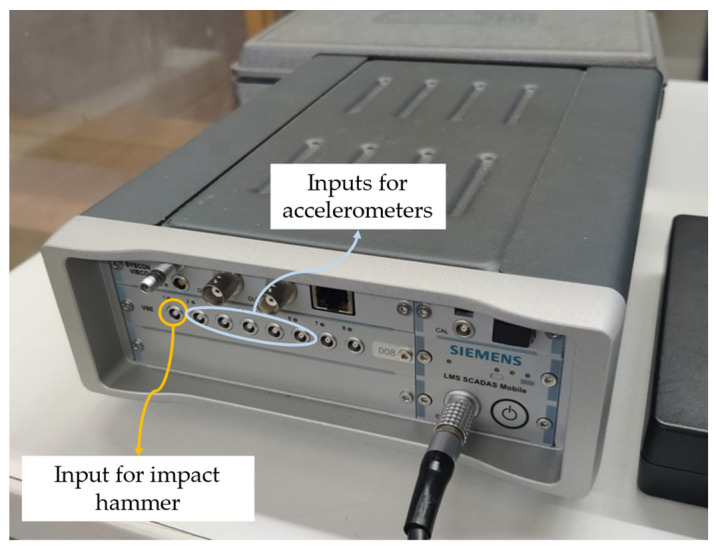
Connection configuration for the measurement system (LMS SCADAS Mobile).

A total of 5 accelerometers (PCB 352C33, PCB Piezotronics) were employed; hence, 5 simultaneous responses were measured, and then the accelerometers were positioned in the consequent measurement points. Four measurements were performed to cover the whole specimen, as reported in Figure 4. The input force was used as a trigger, to record the transient and the damped dynamical response. Each acquisition lasted 0.25 s; the frequency range considered was 0–4096 Hz, i.e., sampling frequency equal to 8192 Hz and 1024 spectral lines (with a frequency resolution of 4 Hz); 5 repeated acquisitions were performed on each measurement point and the average was computed to increase the Signal-to-Noise Ratio (SNR). The LMS SCADAS Mobile (Siemens, Munich, Germany) was exploited as an acquisition system; the connection configuration is reported in Figure 5.

### 2.1. Modal Analysis, Modal Curvatures Computation, and Damage Indices Definition

The modal analysis was conducted through the LMS SCADAS Mobile proprietary software, i.e., the Simcenter Testlab Impact testing module. In particular, the acceleration signals were analysed in relation to the force given by the impact hammer (measured through the embedded load cell) to obtain the Frequency Response Functions (FRFs), which in turn allow to compute the modal parameters of the structural element, hence representing an important tool for damage identification [58].

Data processing allows to visualisation of the vibration distribution on the specimen under test, hence it is possible to identify the areas most subjected to vibration or with discontinuities that could be related to the presence of damages (but also to the presence of embedded sensors). Observing the response signal in the time domain, it is possible to notice an amplification of the excitation when the frequency is close to the structure natural frequency (in correspondence with which the amplitude is maximum). Indeed, the FRF behaves like a filter related to the input force: it amplifies the force at the resonance frequency, whereas it decreases it at antiresonance. Modal shapes can be identified for the different resonance frequencies; the complexity of vibrational modes increases with frequency. It is worth underlining that no displacements are present at nodal lines, and this can be detrimental if we want to exploit vibrational analysis to detect damages close to nodal lines.

Mode shapes are derived through experimental modal analysis based on interpolation methods. In particular, the FRF in terms of acceleration, i.e., the Inertance, was considered. The vibration maps are obtained from the FRFs evaluated as estimators in the frequency domain, according to (1):

(1) $$H_{1}\left(\omega \right)=\frac{S_{fv}\left(\omega \right)}{S_{ff}\left(\omega \right)}$$

where:

- $S_{fv}\left(\omega \right)$ is the cross-spectrum between the vibration acceleration (i.e., accelerometer signal) and the force (i.e., load cell signal).
- $S_{ff}\left(\omega \right)$ is the auto-spectrum of the input force.

The vibrations map is obtained by plotting the amplitude of the H_1_ function in correspondence with the structure resonances. The Polymax algorithm was used for this aim; it estimates the modal parameters in the frequency domain depending on the interpolation of FRFs through fractional polynomial functions.

Hence, diverse strategies were implemented for the comparison between intact and damaged conditions.

At first, changes in terms of both natural vibration frequency (*f_n_*) and loss factor (*η*) were computed, as reported in (2) and (3), respectively.

(2) $$∆f_{n}=(f_{n}^{td}-f_{n}^{t0})/f_{n}^{t0}\times 100\;$$

where:

- $f_{n}^{t0}$ is the natural frequency at *t*0 (intact specimen).
- $f_{n}^{td}$ is the natural frequency at different test times (damaged specimen, i.e., *t*1, *t*2, and *t*3).

(3) $$∆\eta =(\eta^{td}-\eta^{t0})/\eta^{t0}\times 100\;$$

where:

- $\eta^{t0}$ is the loss factor at *t*0 (intact specimen).
- $\eta^{td}$ is the loss factor at different test times (damaged specimen, i.e., *t*1, *t*2, and *t*3).

Also, the MAC between mode shapes was evaluated at each test time (*tj*) related to damaged element with respect to *t*0 (i.e., intact element) on the same specimen and for the same mode. Indeed, the MAC is expected to decrease with damage occurrence.

The subsequent analyses were performed exclusively on the I mode shape of the analysed beams (excluding rigid-body and II mode shape); indeed, in practice, the modal curvature can be computed effectively only for low-order mode shapes [22], given the limited number of measurement points (i.e., 20 DOFs in the present study).

**Figure 6:**
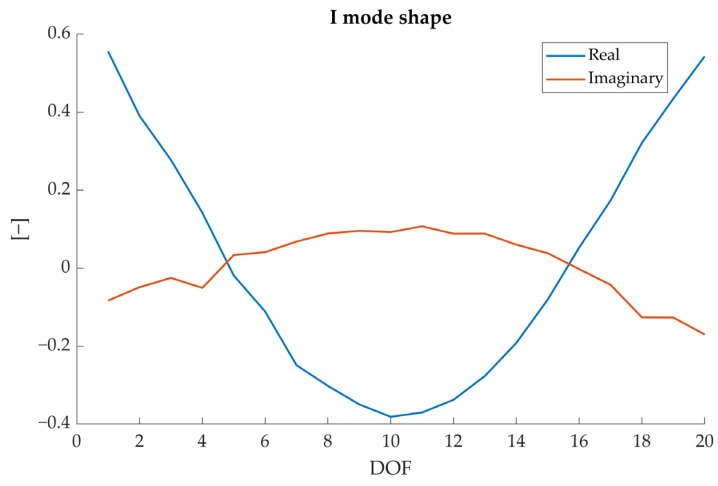
Real (blue) and imaginary (red) parts of the I mode shape (specimen A).

Modal curvatures ($\varphi^{\prime \prime }$) were computed as the second derivative of the related mode shapes; being the latter complex, also the former will be. However, the main informative content is in the real part of the signal, as it is possible to verify in the example reported in Figure 6.

Given the noise typical of experimental data, plus the discrete measurement points, at first, data were oversampled (i.e., the number of points in the signal was increased, with an oversampling factor of ×5 used to increase the original sample rate) in order to have more points on for the computation of modal curvature (since experimental data are acquired on discrete points and the error increases with the distance between two consecutive points [45]). Hence, the central difference method was used to approximate the second derivative of the mode shape, since this method works well for smooth signals, as those obtained increasing the number of the points in the signal.

Then, the cuspid caused by the damage occurrence was evidenced through CWT-based analysis; curvatures were interpolated considering 200 points and Equation (4) was used to extract the real part of the mode shape curvature, being the real part of mode shape more informative [47].

(4) $$\varphi_{comb}^{\prime \prime }=\left|\varphi^{\prime \prime }\right|\times cos\left(∠\varphi^{\prime \prime }\right)$$

where:

- $\left|\varphi^{\prime \prime }\right|$ is the modulus of the modal curvature ($\varphi^{\prime \prime }$)
- $∠\varphi^{\prime \prime }$ is the phase of the modal curvature ($\varphi^{\prime \prime }$)

The continuous wavelet transform essentially makes a convolution of the input signal with functions derived from a mother wavelet (continuous in both time and frequency domains) that is scaled and translated in time. In this study the *cwt* function of MATLAB^®^ (R2023b) was employed; it uses the Morse wavelet. The symmetry (γ) and the time-bandwidth product parameters were set at 2 and 2.5, respectively, empirically selecting them to highlight the curvature formation in the mode shape. The absolute value obtained from this process can be plotted in function of time and frequency, obtaining the so-called scalogram. Then, the image was binarized using the global threshold method; hence, a binary image was created exploiting Otsu’s method [59], which creates a global threshold minimizing the intraclass variance of black/white pixels subjected to the threshold. Then, the area of the high-valued pixels was computed, and the values obtained at the different test times were compared.

Finally, three different damage indices were calculated. *DI_curv_*, as reported in (5), is the sum of the absolute differences in modal curvature between each test time related to the damaged specimen with respect to the intact specimen (assessed at *t*0), where each difference is normalized with respect to the absolute value of the curvature maximum value at *t*0.

(5) $${DI}_{curv}=\sum_{i=1}^{N}\frac{\left|{\varphi^{\prime \prime }}_{tx}-{\varphi^{\prime \prime }}_{t0}\right|}{max\left|{\varphi^{\prime \prime }}_{t0}\right|}$$

where:

- ${\varphi^{\prime \prime }}_{tx}$ is the modal curvature computed at the t_x_ test time (i.e., *t*1, *t*2, and *t*3).
- ${\varphi^{\prime \prime }}_{t0}$ is the modal curvature computed at *t*0 (intact specimen).

Substantially, this index recalls that proposed by Pranno et al. [22] (i.e., CDF), normalizing it with respect to $max\left|{\varphi^{\prime \prime }}_{t0}\right|.$ However, in this study, only the I-mode shape was considered.

A second damage-related index, *DI_CWT_*, was defined based on CWT-based analysis. At first, the image obtained from CWT computation was binarized according to an automated threshold defined according to Otsu’s method [59]. Then, the area of the high CWT coefficients in the binarized image was computed and the value was normalized with respect to the area obtained for *t*0, as reported in (6).

(6) $${DI}_{CWT}=\frac{{\left.\sum_{i=1}^{N}\left(pixel=1\right)\right|}_{tx}}{{\left.\sum_{i=1}^{N}\left(pixel=1\right)\right|}_{t0}}$$

While *DI_curv_* is expected to increase with damage, since changes in modal curvature will be more significant, *DI_CWT_* should decrease (i.e., smaller high pixel area).

A third damage index, *DI_global_*, was defined, by combining the previous ones, as reported in (7).

(7) $${DI}_{global}=\frac{{DI}_{curv}}{{DI}_{CWT}}$$

This index is expected to increase with damage and its importance since the numerator should increase while the denominator should decrease with damage occurrence and also with increasing severity. In this way, both changes in modal curvatures inferred from CWT-based analysis are included, making this index more robust.

### 2.2. Sensitivity Analysis to Data Processing Parameters

A sensitivity analysis was performed to investigate the sensitivity of the results in terms of damage indices to some data processing parameters, in particular:

- Interpolation smoothing factor.
- Oversampling factor.

The first factor is involved in the cubic spline interpolation employed before the computation of modal curvature. Indeed, without interpolating the mode shapes the discontinuity due to the presence of damage would be more evident; on another hand, the presence of noise in experimental data would provide a bad-quality baseline signal (i.e., modal curvature related to the intact specimen). Hence, a compromise is needed between the aim of obtaining good quality data and the desire to clearly detect the damage-related discontinuity. In fact, the literature states that modal curvature computation is effective only with smooth signals [45]. Regarding oversampling, the step is needed to have a lower distance between points, thus reducing the noise due to limited experimental data [45], which in this way are increased in post-processing by multiplying the original sample rate by a certain factor.

Therefore, a sensitivity analysis was performed on specimen data (i.e., specimen A) and the tested values for the two considered variables are reported in Table 2. The effect of the selection of different processing parameters was evaluated in terms of damage-related indices.

## 3. Results and Discussion

The flexural strength measured on the dedicated specimens was equal to 12 kN. However, due to the intrinsic variability of concrete, the tested beams conveyed different fracture loads (13.5 ± 0.89, reported as mean ± standard deviation), as illustrated in Table 3.

### 3.1. Modal Parameters

The results from the modal analysis are reported in terms of natural vibration frequency (Table 4) and loss factor (Table 5) obtained for each test time (i.e., *t*0, *t*1, *t*2, and *t*3), and MAC values obtained correlating the mode shapes estimated for the three loading tests to the mode shapes estimated in the undamaged condition (i.e., *t*0) on the same specimen (Table 6).

**Figure 7:**
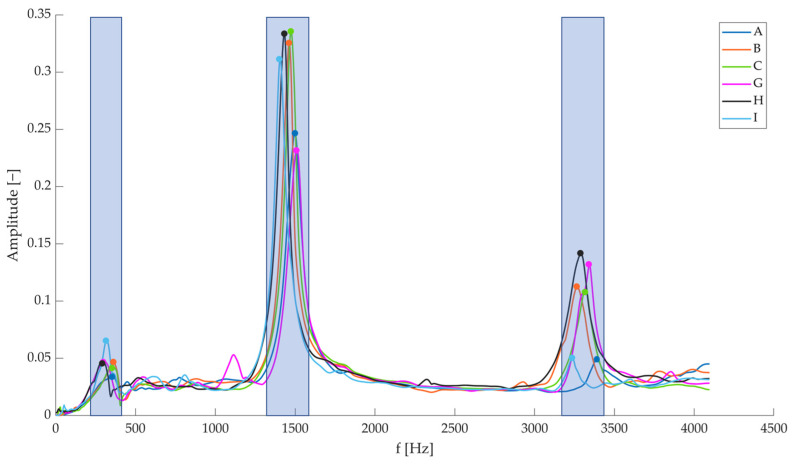
Sum FRFs obtained on the different specimens at *t*0 (intact beams). The three blue bands indicate the three considered mode shapes, namely rigid-body mode shape (**left**), I mode shape (**centre**), and II mode shape (**right**). The circular markers are related to their natural vibration frequencies.

At first, the FRFs obtained in terms of Inertance were analysed to identify the low-frequency modes; in this range rigid-body mode, I, and II mode shapes were identified, as reported in Figure 7 for *t*0 test time (undamaged specimens). It is possible to notice a certain variability in the dynamic behaviour of the tested specimens. If the natural vibration frequency of the I mode shape is considered (I is the most evident one), a variability of approximately 100 Hz can be evidenced. Hence, if a variation of that order is observed, it cannot be attributed to external factors (e.g., damage occurrence), since it falls within the specimen variability range. No significant differences can be evidenced between sensorized (i.e., A, B, and C) and not-sensorized (i.e., G, H, and I) specimens.

**Figure 8:**
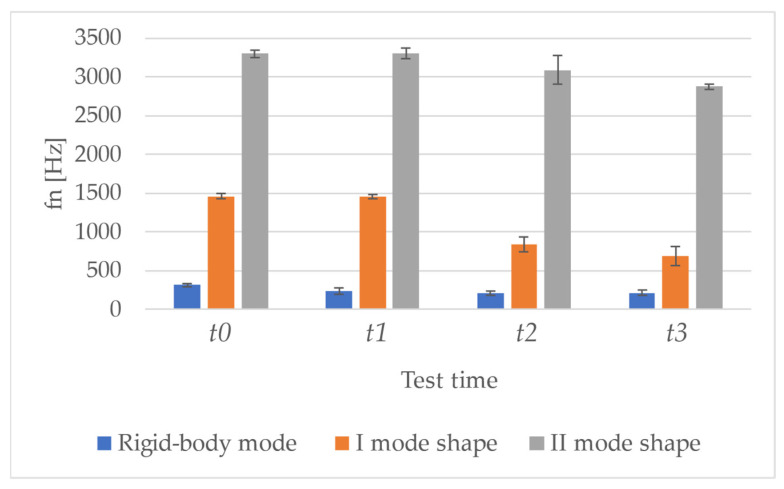
Natural vibration frequencies are reported as mean ± standard deviation at each test time for all the specimens.

**Figure 9:**
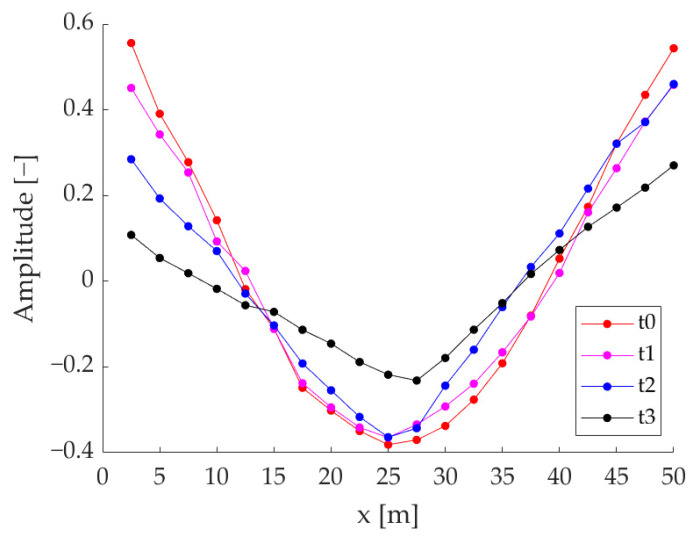
Example of I mode shape with cuspid formation due to crack (*t*2 and *t*3 test times)—specimen A.

Changes caused by cracking phenomena (Figure 8) are more easily visible in the I mode shape, whose natural vibration frequency is reduced by (42.12 ± 7.91)% at *t*2 (first crack formation) and practically halved at *t*3, with a decrease of (52.61 ± 8.20)%. It is possible to recognize a cuspid in the mode shape (Figure 9), highlighting the presence of damage in the specimen mid-line. This feature can be further evidenced through the computation of modal curvature, as reported in Section 3.2. Concerning the II mode shape, it is worth noting that at *t*0 its natural vibration frequency was probably at higher frequencies; then, with the occurrence of cracks, the natural frequency decreased, re-entering the observed frequency band (i.e., 4096 Hz). However, that mode presents a nodal line in correspondence with the beam mid-line, hence the damage is hardly identifiable. For this reason, the remaining part of the damage-identification procedure was focused on the I mode shape.

Being the vibrational tests conducted after the load removal, it should be stressed that the differences in results at *t*2 and *t*3 could be not always very evident since the crack partially re-closes before the impact test execution.

**Figure 10:**
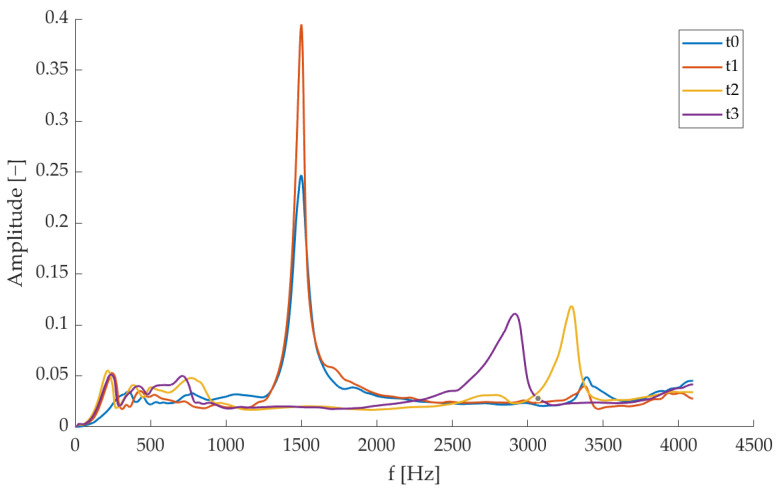
Sum FRFs at different test times (Specimen A).

Regarding the first crack formation (i.e., *t*2), changes in the natural vibration frequency (Figure 8) are equal to (–42.12 ± 7.91)% for the I mode shape; this is also visible from the Sum FRFs (Figure 10). When the crack aperture is widened until approximately 1 mm, the changes achieve (−52.61 ± 8.20)%; in particular, it can be noticed that the natural vibration frequency passes from a mean value of 1461 Hz (±37 Hz) at *t*0 to 843 Hz (±97 Hz) at *t*2 and to 693 Hz (±126 Hz) at *t*3. However, the results are not consistent among all the tested specimens, since vibrational tests were performed after having removed the external load, so the crack has the possibility of partially closing, as mentioned above. Even if beyond the scope of this paper, it is worth underlining that the crack aperture was assessed through a previous measurement procedure based on vision techniques [60] and the measured values were in the range of 0.3–0.6 mm; this confirms the partial crack closure after the load removal. Furthermore, being the structure reinforced, the damage is diffused.

**Figure 11:**
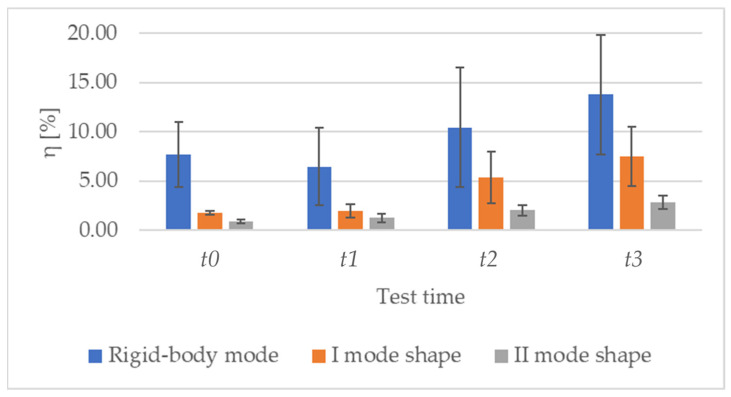
Loss factor values are reported as mean ± standard deviation at each test time for all the specimens.

Concerning the loss factor (Table 4), given the presence of the crack and therefore the enhancement of the vibration loss through the propagation into the fracture itself, it is possible to observe an increasing trend with test times (Figure 11). In particular, η value passes from (7.69 ± 3.35)% at *t*0 to (13.77 ± 6.11)% at t3 for rigid-body shape, from (1.76 ± 0.20)% at *t*0 to (7.52 ± 3.01)% at *t*3 for I mode shape, and from (0.88 ± 0.17)% at *t*0 to (2.82 ± 0.67)% at *t*3 for II mode shape. However, it is worth noting the high variability among specimens, but this is in line with what is expected, also from the literature [36].

**Figure 12:**
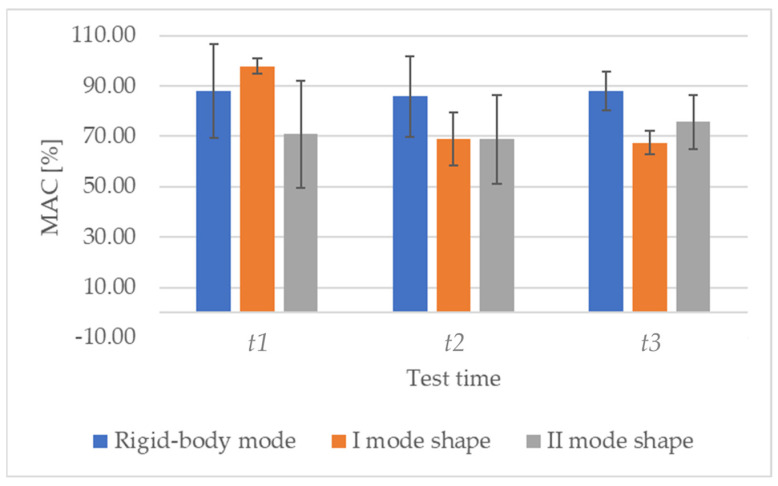
MAC values are reported as mean ± standard deviation at *t*1, *t*2, and *t*3 test times with respect to *t*0 for all the specimens.

Analysing the MAC values (Table 6), a decrease in correlation at *tx* with respect to *t*0 test time (i.e., same specimen in undamaged conditions) is expected over test times, since the formation of cracks makes the mode shapes change. Indeed, this behaviour is well evident only for the I mode shape, since both rigid-body shape and II mode shape show compatible values, for example, at *t*1 and *t*2. It is a different matter for the comparison between t2 and t3, given that the analyses were performed after having removed the load, which let the crack partially close, as mentioned above. The mean trends and their variability are reported in Figure 12.

Since the modification is more evident for the I mode shape (e.g., the rigid-body shape proves to be insensitive to damages, showing no deflection), the successive analyses (regarding both modal curvatures and CWT-base analyses) were focused on it; indeed, if the mode shapes are observed, at *t*3 it is clear that the beams are broken and vibrate as two quasi-separate objects and this behaviour partially shows at *t*2 (Figure 9).

### 3.2. Modal Curvatures and CWT-Based Analysis

The computation of modal curvatures allows to highlight of the cuspid formation in correspondence with the damage; it is worth underlining that the main crack forms in the specimen centerline, hence the damage is hardly identifiable from mode shapes presenting a nodal line in this area (e.g., II mode shape). This further justifies the choice of performing this analysis only on I mode shape (excluding rigid-body mode).

**Figure 13:**
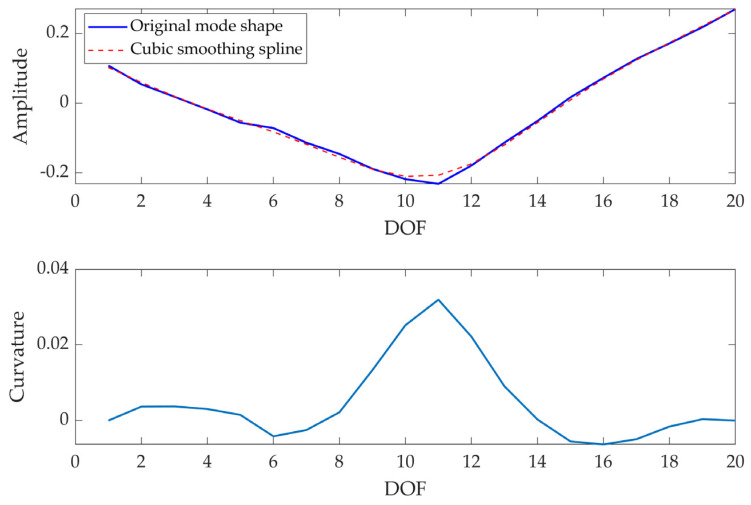
Modal curvature computation for I mode shape: mode shape (raw and smoothed) (**top**), and modal curvature (**bottom**)—(Specimen A, test time: *t*3).

**Figure 14:**
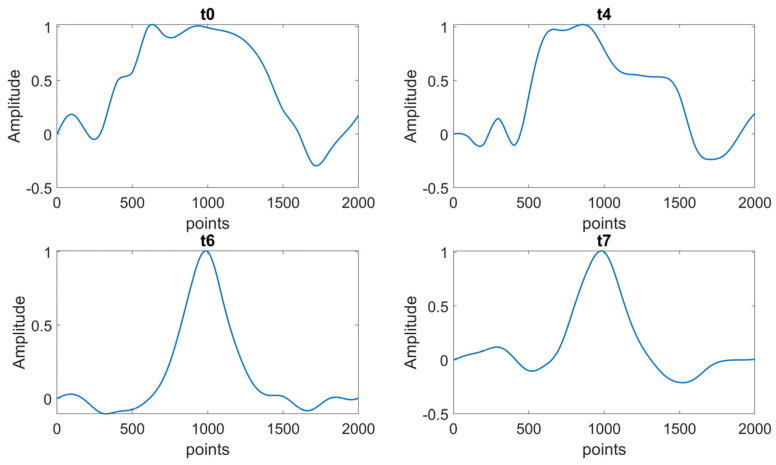
Comparison of I modal curvature computed at each test time (i.e., *t*0, *t*1, *t*2, and *t*3—Specimen A)—2000 points obtained through interpolated curvatures.

**Figure 15:**
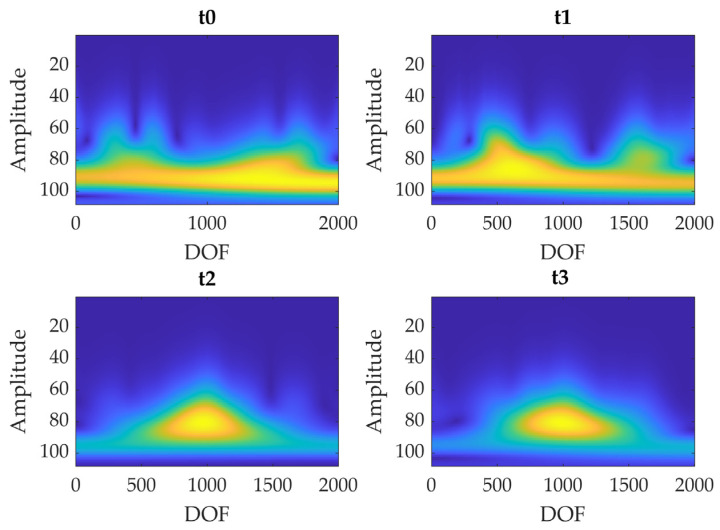
Comparison of the CWTs scalograms of I modal curvature computed at each test time (i.e., *t*0, *t*1, *t*2, and *t*3—Specimen A).

**Figure 16:**
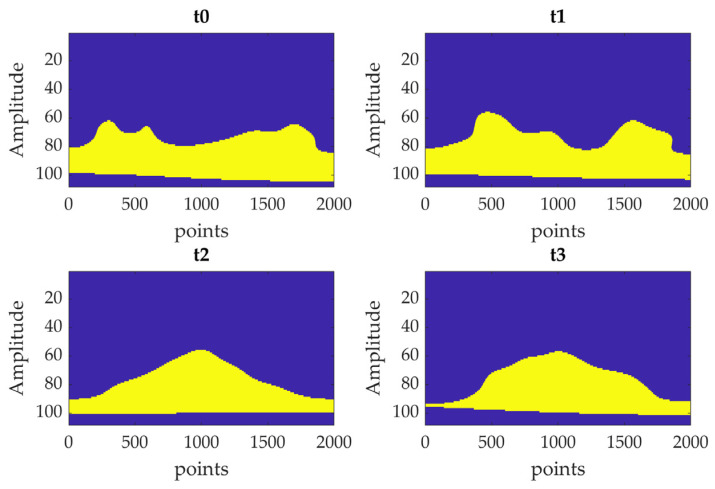
Comparison of the binarized CWTs scalograms of I modal curvature computed at each test time (i.e., *t*0, *t*1, *t*2, and *t*3—Specimen A).

An example of the modal curvature computation, referred to as specimen A, is reported in Figure 13; the preliminary smoothing of the mode shape (through a cubic spline interpolation with a smoothing factor of 0.4) allows to obtain a less noisy curvature. The comparison among curvatures obtained at different test times is reported in Figure 14 (where curvatures are interpolated with a factor of 100, hence 2000 points are obtained); the cuspid appears with the crack formation at *t*2 and the morphology is clearly distinguishable from *t*0 (i.e., undamaged conditions). The application of CWT makes its objective identification easily feasible, as can be observed in Figure 15; indeed, the binarization of the image (Figure 16) leads to the definition of an area shape immediately referring to the cuspid and, hence, to the damage occurrence.

### 3.3. Damage Related Indices

**Figure 17:**
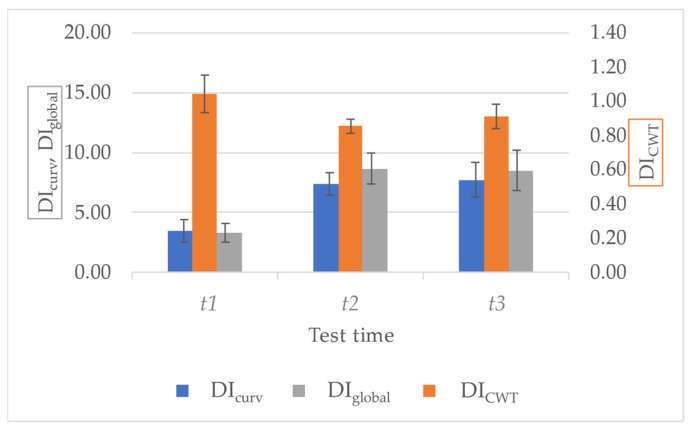
Damage-related indices were reported as mean ± standard deviation at each test time. Note: The left vertical axis refers to *DI_curv_* and *DI_global_*, whereas the right vertical axis is related to *DI_CWT_* for all the specimens.

As a synthetic way to identify and represent the damaged status of a cement-based structural element, the authors evaluated different indices, namely *DI_curv_*, *DI_CWT_*, and *DI_global_* (see Section 3 for details). The results obtained on all the tested specimens for each test time are reported in Table 7 and are summarized in Figure 17; it can be observed that the values obtained for *t*2 and *t*3 test times fall in compatible measurement ranges, meaning that the two conditions cannot be distinguished. This is probably linked to the fact that the vibrational analyses were performed after the load removal, letting the crack partially close. On the contrary, there is a significant variation between the indices obtained at *t*1 (i.e., 90% of fracture load) and the values after damage occurrence (i.e., at *t*2 and *t*3). The first crack formation (i.e., *t*2) can be promptly detected, signalling an alteration of the dynamic behaviour of the element; this proves that the proposed assessment strategy is effective and adequately sensitive to the occurrence of damage. Hence, the results confirm the possibility of discriminating between intact (i.e., *t*1) and damaged (i.e., *t*2 and *t*3) conditions, but further investigations are needed to distinguish among different levels of damage (e.g., between *t*2—crack formation—and *t*3—crack aperture of 1 mm—conditions).

### 3.4. Sensitivity Analysis

**Figure 18:**
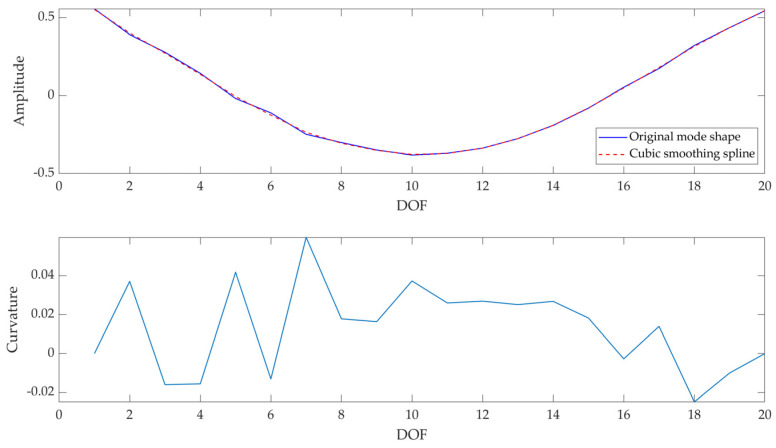
Modal curvature computation for I mode shape: mode shape (raw and smoothed) (**top**), and modal curvature (**bottom**)—(Specimen A, test time: *t*0), smoothing factor: 0.9.

**Figure 19:**
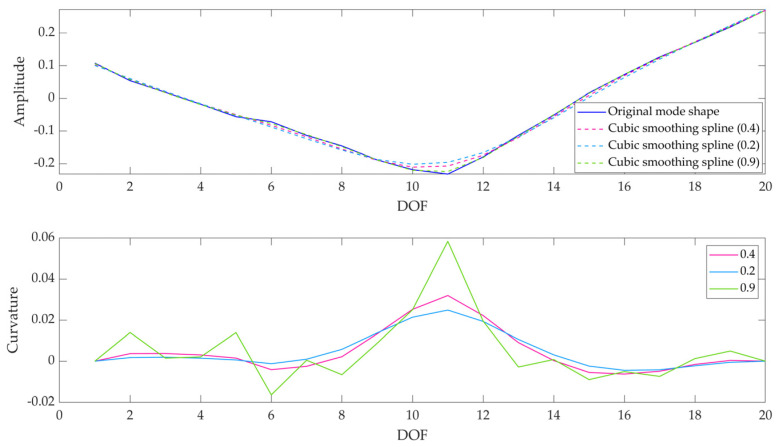
Modal curvature computation for I mode shape: mode shape (raw and smoothed) (**top**), and modal curvature (**bottom**)—(Specimen A, test time: *t*7), comparison among different smoothing factors.

The results in terms of the sensitivity of damage-related indices to data processing variables, namely smoothing factor, and oversampling factor, are reported in Table 8. It is worth noting that the sensitivity analysis has been performed separately on the two variables. It is possible to observe that the change of smoothing factor has a significant effect on all the damage-related indices (in case of *DI_curv_*, with an almost constant sensitivity, varying in the range of 2.2–2.6 depending on the test time, as it can be deduced from Table 8); when it is halved (passing from 0.4 to 0.2), *DI_curv_* decreases of approximately 26%, *DI_CWT_* increases of about 16%, and *DI_global_* decreases of about 36% at t3 test time. The *DI_CWT_* index trend is no longer monotonically decreasing as expected since the smoothing operation is too aggressive and the cuspid is no more identifiable. On the other hand, considering the softest smoothing operation (i.e., smoothing factor equal to 0.9), it is possible to notice that this is not sufficient to obtain a good quality baseline curvature at *t*0 (Figure 18). The damage is no longer identifiable through none of the indices (their trends are opposed compared to the expected ones)—even if considering only *t*3 test time the cuspid would be more evident (but at the expense of signal quality, especially at *t*0). Therefore, a smoothing factor equal to 0.40 can be considered adequate for our purposes (a comparison can be observed in Figure 19). Concerning the oversampling factor, its effect is slightly on *DI_CWT_* and, hence, *DI_global_* indices. No significant changes can be evidenced, hence a ×5 oversampling factor can be considered a good compromise to have a good quality signal and limited computational load at the same time.

## 4. Conclusions

The experimental modal analysis performed in this study allowed us to identify the crack formation in the tested scaled concrete beams; in particular, the damage occurrence causes (i) a decrease in natural vibration frequency and (ii) an increase of the loss factor (since the element rigidity decreases). Moreover, a decrease in terms of MAC between the mode shapes estimated for damaged samples and the mode shapes estimated for the corresponding undamaged samples can be observed with the damage occurrence, due to the change of the mode shapes morphology. The variations of both modal parameters and mode shapes can be highlighted through the computation of the related modal curvatures (after proper signal pre-processing to enhance SNR, considering the sensitivity of results towards data processing parameters, in particular, smoothing factor); moreover, specific processing techniques, namely CWT, can help in objectively identifying the formation of discontinuities (e.g., cuspid) in the modal curvatures, which are characteristic of the induced damage. Finally, synthetic damage indices can be used to provide a global indication of the health status of a cement-based element or structure.

In practical in-field applications, if the measurements at *t*0 (undamaged conditions) are not available, a validated numerical model could provide the baseline modal parameters of the structure. For example, a comparison between the experimental curvature and the theoretical one (numerically obtained) could be carried out to evaluate the presence of possible damages in the structure. Indeed, the diagnostic methods based on a comparison with baseline data are less tricky, whereas analysing only experimental curvatures may require more complex computational methods to identify eventual damages. Specific thresholds should be considered since they are problem-dependent values. Moreover, it is worth underlining the importance of data quality, since noisy data hinder the computation of reliable modal curvatures; smoothing techniques can be applied before deriving the signal to this aim, but this could mask also the discontinuities linked to the damage occurrence. Hence, a good compromise between the use of filtering and smoothing techniques to enhance SNR and the need to preserve damage-related discontinuities should be pursued. Nevertheless, data processing techniques are pivotal when dealing with experimental data, which are inevitably affected by noise and are more demanding compared to numeric results derived from synthetic models. Data processing parameters related to smoothing and oversampling techniques enhancing the signal quality can influence the final results (as evidenced by the sensitivity analysis performed in this work), hence they should be properly selected. Moreover, it is important to stress the fact that the considered frequency band directly influences the mode shapes that can be investigated, which will be inevitably less than those observable in the case of numerical models.

Finally, in the future more indices (possibly inferred from multidomain signals) could be combined to increase the method sensitivity to damages, also thanks to data fusion techniques. In addition, the suitability of the method for regular structural monitoring could be evaluated; in this context the use of Laser Doppler Vibrometry (LDV) could be evaluated for in-field applications, to enable the vibrational analysis even in operating conditions and in (near) real-time (e.g., during the application of a load). In this way, the proposed strategy could become a monitoring technique, without the limits of single (or at most periodic) inspections.

## Figures and Tables

**Table 1:** Mix-design of concrete specimens.

Cement [kg/m^3^]	Water [kg/m^3^]	Air [%]	Sand [kg/m^3^]	Intermediate Gravel [kg/m^3^]	Coarse Gravel [kg/m^3^]	RCF [kg/m^3^]	BCH [kg/m^3^]
470.0	235.0	2.5	795.0	321.0	476.0	0.9	10.0

**Table 2:** Sensitivity analysis parameters: values considered for smoothing and oversampling factors.

Variable	Values
Smoothing factor	0.20
	0.40
	0.90
Oversampling factor	×2
	×5
	×10

**Table 3:** Fracture loads under flexure for each tested specimen.

Specimen	Fracture Load [kN]
A	13.1
B	13.1
C	15.2
G	13.8
H	13.5
I	12.3

**Table 4:** Natural vibration frequency values for each tested specimen (A, B, C, G, H, and I) at each test time (*t*0, *t*1, *t*2, and *t*3) and related percentage variations with respect to undamaged condition (*t*0).

Specimen	Test Time	*f_n_* (Δ*f_n_* [%]) [Hz]		
		Mode Shape		
		Rigid-Body	I	II
A	*t*0	341 (-)	1487 (-)	3371 (-)
	*t*1	237 (−30.44)	1488 (−0.07)	3383 (−0.35)
	*t*2	218 (−36.12)	796 (−46.47)	3265 (−3.14)
	*t*3	210 (−38.48)	715 (−51.92)	2888 (−14.34)
B	*t*0	329 (-)	1446 (-)	3273 (-)
	*t*1	294 (−10.64)	1442 (−0.34)	3295 (−0.67)
	*t*2	249 (−24.20)	748 (−48.30)	3172 (−3.09)
	*t*3	258 (−21.64)	481 (−66.73)	2871 (−12.28)
C	*t*0	343 (-)	1467 (-)	3315 (-)
	*t*1	285 (−16.91)	1462 (−0.33)	3331 (−0.48)
	*t*2	203 (−40.82)	794 (−45.88)	3170 (−4.37)
	*t*3	259 (−24.52)	597 (−59.33)	2884 (−13.00)
G	*t*0	289 (-)	1519 (-)	3338 (-)
	*t*1	235 (−18.69)	1487 (−2.10)	3330 (−0.26)
	*t*2	199 (−31.04)	767 (−49.50)	2687 (−19.52)
	*t*3	212 (−26.48)	870 (−42.73)	2861 (−14.31)
H	*t*0	303 (-)	1441 (-)	3280 (-)
	*t*1	159 (−47.47)	1425 (−1.10)	3319 (−1.18)
	*t*2	160 (−47.19)	951 (−33.98)	3127 (−4.66)
	*t*3	181 (−40.36)	786 (−45.41)	2942 (−10.31)
I	*t*0	300 (-)	1403 (-)	3224 (-)
	*t*1	215 (−28.52)	1412 (−0.63)	3173 (−1.58)
	*t*2	206 (−31.41)	1002 (−28.59)	3142 (−2.54)
	*t*3	175 (−41.79)	708 (−49.55)	2829 (−12.25)

**Table 5:** Loss factor (*η*) values for each tested specimen (A, B, C, G, H, and I) at each test time (*t*0, *t*1, *t*2, and *t*3) and related percentage variations (Δ*η*) with respect to undamaged condition (*t*0).

Specimen	Test Time	*η* (Δ*η* [%]) [%]		
		Mode Shape		
		Rigid-Body	I	II
A	*t*0	10.76 * (-)	1.70 (-)	0.69 (-)
	*t*1	2.48 (−76.99 *)	1.15 (−32.35 *)	0.86 (24.35)
	*t*2	3.99 (−62.96 *)	3.69 (117.09)	1.17 (69.42)
	*t*3	12.35 (14.80)	3.98 (134.12)	2.23 (223.37)
B	*t*0	9.45 (-)	1.98 (-)	0.70 (-)
	*t*1	10.31 (9.05)	2.67 (35.35)	0.95 (34.86)
	*t*2	17.45 (84.57)	9.23 (367.19)	1.42 (102.19)
	*t*3	20.85 (120.52)	11.44 (479.27)	2.25 (219.69)
C	*t*0	5.56 (-)	2.03 (-)	0.91 (-)
	*t*1	6.15 (10.55)	2.27 (11.97)	1.41 (54.93)
	*t*2	10.40 (87.05)	6.30 (210.58)	2.54 (179.82)
	*t*3	11.36 (104.25)	9.14 (350.61)	4.11 (353.01)
G	*t*0	9.34 (-)	1.59 (-)	0.78 (-)
	*t*1	9.34 (0.00)	2.00 (26.14)	0.78 (0.00)
	*t*2	14.75 (57.96)	7.76 (389.43)	2.22 (186.60)
	*t*3	19.12 (104.79)	9.56 (502.87)	3.05 (293.02)
H	*t*0	1.16 * (-)	1.46 * (-)	1.01 (-)
	*t*1	0.22 (−81.45 *)	0.84 (−42.12 *)	1.55 (52.82)
	*t*2	0.85 (−26.72)	1.73 (18.82)	2.67 (163.97)
	*t*3	2.38 (105.33)	3.11 (113.36)	2.30 (127.47)
I	*t*0	9.87 (-)	1.79 (-)	1.17 (-)
	*t*1	10.31 (4.51)	2.68 (49.76)	1.99 (70.08)
	*t*2	15.23 (54.29)	3.45 (92.79)	2.05 (75.21)
	*t*3	16.56 (67.78)	7.90 (341.55)	2.95 (152.13)

* Outliers.

**Table 6:** MAC values for each tested specimen (A, B, C, G, H and I) considering different test times (i.e., *t*1, *t*2, and *t*3) with respect to *t*0 (i.e., undamaged conditions).

Specimen	Test Time	MAC [%]		
		Mode Shape		
		Rigid-Body	I	II
A	*t*0	93.81	90.97	29.19
	*t*1	93.14	78.19	59.84
	*t*2	87.50	72.85	57.59
	*t*3	98.63	98.63	89.64
B	*t*0	94.44	64.21	84.20
	*t*1	85.31	66.86	89.10
	*t*2	97.47	99.67	82.71
	*t*3	93.60	50.40	86.58
C	*t*0	92.14	60.88	82.65
	*t*1	97.13	99.31	84.85
	*t*2	84.74	64.44	34.35
	*t*3	94.96	74.40	83.07
G	*t*0	46.34	99.36	81.35
	*t*1	50.94	80.74	72.58
	*t*2	72.75	64.52	75.62
	*t*3	94.78	99.41	57.18
H	*t*0	97.81	76.38	75.40
	*t*1	95.61	65.20	66.77
	*t*2	93.81	90.97	29.19
	*t*3	93.14	78.19	59.84
I	*t*0	87.50	72.85	57.59
	*t*1	98.63	98.63	89.64
	*t*2	94.44	64.21	84.20
	*t*3	85.31	66.86	89.10

**Table 7:** Damage-related indices for each tested specimen (A, B, C, G, H, and I) at each test time (*t*1, *t*2, and *t*3).

Specimen	Test Time	Damage Indices		
		*DI_curv_*	*DI_CWT_*	*DI_global_*
A	*t*1	4.98	1.01	4.94
	*t*2	8.74	0.83	10.53
	*t*3	8.66	0.82	10.59
B	*t*1	3.93	1.17	3.36
	*t*2	7.49	0.89	8.44
	*t*3	7.82	0.90	8.69
C	*t*1	2.61	0.96	2.73
	*t*2	8.35	0.81	10.33
	*t*3	8.90	1.00 *	8.92 *
G	*t*1	2.13	0.87	2.44
	*t*2	6.03 *	0.82	7.37 *
	*t*3	5.03	0.85 *	5.93
H	*t*1	3.61	1.08	3.36
	*t*2	7.07	0.92	7.65
	*t*3	9.13	0.90	10.16
I	*t*1	3.64	1.17	3.10
	*t*2	6.57	0.86 *	7.65 *
	*t*3	7.49	0.89	8.44

* Outliers.

**Table 8:** Results of sensitivity analysis.

Variable	Values	Test Time	Damage Indices		
			*DI_curv_*	*DI_CWT_*	*DI_global_*
Smoothing factor (with oversampling factor ×5)	0.20	*t*1	2.88	1.01	2.85
		*t*2	6.82	0.94	7.29
		*t*3	6.39	0.95	6.75
	0.40	*t*1	4.98	1.01	4.94
		*t*2	8.74	0.83	10.53
		*t*3	8.66	0.82	10.59
	0.90	*t*1	8.09	0.61	13.22
		*t*2	11.26	1.01	11.17
		*t*3	11.36	1.28	8.68
Oversampling factor (with smoothing factor 0.4)	×2	*t*1	4.98	1.02	4.85
		*t*2	8.74	0.85	10.34
		*t*3	8.66	0.81	10.69
	×5	*t*1	4.98	1.01	4.94
		*t*2	8.74	0.83	10.53
		*t*3	8.66	0.82	10.59
	×10	*t*1	4.98	1.01	4.92
		*t*2	8.74	0.84	10.44
		*t*3	8.66	0.82	10.51

## Data Availability

The data presented in this study are available on request from the corresponding author.
